# Inclusion of Evidence-Based Breast Cancer Control Recommendations and Guidelines in State Comprehensive Cancer Control Plans

**DOI:** 10.5888/pcd17.200046

**Published:** 2020-10-15

**Authors:** Mehrnoosh Soori, Elizabeth A. Platz, Norma Kanarek

**Affiliations:** 1 Department of Epidemiology, Johns Hopkins Bloomberg School of Public Health, Baltimore, Maryland; 2 Sidney Kimmel Comprehensive Cancer Center at Johns Hopkins, Baltimore, Maryland; 3 Department of Environmental Health and Engineering, Johns Hopkins Bloomberg School of Public Health, Baltimore, Maryland

## Abstract

**Introduction:**

Each US state, territory, and tribe/tribal organization is supported by the Centers for Disease Control and Prevention to develop and implement a comprehensive cancer control (CCC) plan. The objective of this study was to inform areas for improvement of those plans.

**Methods:**

To show how CCC plans can be improved, we used the example of breast cancer, which has a long public health history and an established, broad spectrum of prevention and control activities. We evaluated the inclusion of evidence-based breast cancer prevention topics as provided by guidelines from the Centers for Disease Control and Prevention (CDC) and recommendations of the US Preventive Services Task Force (USPSTF) in each state’s CCC plan. From January through March 2019, we downloaded CCC plans from each state and the District of Columbia and abstracted and quantified the content of plans for 1) discussion of data on breast cancer mortality, breast cancer incidence, uptake of mammography; 2) statement of objective to reduce the burden of breast cancer; and 3) review of CDC guidelines and USPSTF recommendations.

**Results:**

The discussion of breast cancer–relevant topics and specification of objectives was incomplete. Of 51 plans, data on breast cancer mortality and incidence and uptake of mammography were reported in 53% (n = 27) to 76% (n = 39) of plans. CDC and USPSTF recommendations for breast cancer–specific interventions were discussed in only 6% (n = 3) to 37% (n = 19) of plans. Discussion of general cancer prevention topics relevant to breast cancer ranged from 10% (n = 5) to 61% (n = 31) of plans.

**Conclusion:**

Our findings inform areas for quality improvement of state CCC plans and may contribute to other areas of public health planning.

**Summary**

**What is already known on this topic?**

State comprehensive cancer control (CCC) plans are supported through national programs in the United States and are written and updated by using consensus strategies.

**What is added by this report?**

Using breast cancer as an example, we describe adherence to national recommendations or guidelines in crafting objectives in state CCC plans.

**What are the implications for public health practice?**

To raise awareness of all that can be done to address the burden of cancer in their state, states need to heed evidence-based recommendations and guidelines and give attention to completeness of objectives in their state CCC plans.

## Introduction

Breast cancer is the most commonly diagnosed cancer and the most common cause of cancer-related death among women in the United States (1). Breast cancer development is attributable to both nonmodifiable (eg, genetic predisposition) and modifiable (eg, reproductive, lifestyle) factors. Modifiable risk factors correlate with a spectrum of interventions available to address reductions in incidence or mortality. Maintaining a healthy weight, being physically active, eating an optimal diet (nutrition) with moderate to no alcohol intake, and breastfeeding may account for future declines in incidence by 29% (2–4). Timely age-specific screening accounts for a 28% to 65% decrease in mortality (5,6). High-risk status has often been determined from nonmodifiable factors (genetic factors and previous benign breast disease) (7,8). For women at high risk of breast cancer, chemoprevention and prophylactic surgery are available as primary prevention strategies (2–4,9).

The Centers for Disease Control and Prevention’s (CDC’s) National Comprehensive Cancer Control Program (NCCCP) funds US states, territories, and tribes/tribal organizations to develop and implement plans to control cancer. CDC recommends state Comprehensive Cancer Control (CCC) plans include evidence-based recommendations and guidelines (10). Accordingly, CDC recommends that state plans, which vary in their content and organization, present valid data from the state’s cancer registry, describe the prevalence of cancer in diverse populations, and provide information on state population demographic characteristics. Plans should also present logically linked, clearly labeled specific, measurable, attainable, relevant and time-phased (SMART) objectives (10).

Because breast cancer has a long history of extensive research supporting policy and program development and a broad spectrum of prevention and control activities, it provides a key test case for determining the quality of CCC plans, and more generally, for studying pitfalls and challenges of cancer prevention and control planning. We evaluated whether CCC plans discussed evidence-based breast cancer prevention topics as described in the most recent CDC guidelines and US Preventive Services Task Force (USPSTF) recommendations (Table 1). Our study objective was to inform areas for quality improvement of state CCC plans by using the example of breast cancer. This study may also inform state planning strategies (eg, SMART objectives) for additional areas of public health.

**Table 1:** General and Breast Cancer Prevention Topics Discussed in the Recommendations of the US Preventive Services Task Force (USPSTF) and Guidelines From the Centers for Disease Control and Prevention (CDC)^a^

Topic	USPSTF	CDC
**Breast cancer burden**		
Mammogram	x	x
**Breast cancer–specific topics**		
Chemoprevention	x	x
Genetic risk assessment, testing, screening	x	x
**General cancer prevention topics that apply to breast cancer**		
Alcohol intake	x	x
Breastfeeding	x	x
Diet/nutrition		x
Healthy weight	x	x
Physical activity		x

a This table was designed by the authors to enable a study of breast cancer–related content in the Comprehensive Cancer Control plans in 50 states and the District of Columbia, January–March 2019.

## Methods

We abstracted information from CCC plans on 3 population-based measures of breast cancer burden, 2 breast cancer–specific topics, and 5 general primary prevention topics that would be included in state CCC plans if CDC and USPSTF recommendations and guidelines had been incorporated (Table 1). We downloaded 51 current CCC plans, from 50 US states and the District of Columbia, from the CDC website (10) from January through March 2019. We did not include in our analysis the CCC plans in US territories because not all territories have a CCC plan.

Every state writes a self-determined CCC plan consistent with CDC plan guidance (10). States select format, priorities, audience, and content of CCC plans. To accommodate this variety, we used a standardized method to abstract and classify from each plan information on breast cancer–related topics occurring in any section in the CCC plan. One member of the research team (M.S.) created a database, abstracted the content, and to ensure accuracy of abstraction, scanned each plan twice.

**Evidence-based breast cancer–related topics in CDC guidelines and USPSTF recommendations.** We reviewed the most up-to-date recommendations and guidelines as of November 30, 2019, on breast cancer control and prevention from USPSTF recommendations (7,8, 11–14) and CDC guidelines (15–22) (Table 1, Table 2) We classified topics by whether they were breast cancer–specific topics, general cancer prevention topics that apply to breast cancer, or measures of breast cancer mortality, incidence, or screening (timely mammogram) prevalence. We defined breast cancer–specific topics as 1) chemoprevention for women at high risk of breast cancer and 2) genetic risk assessment, testing, and screening for breast cancer susceptibility 1 and 2 (*BRCA 1/2*) gene mutations. We defined general cancer prevention topics that applied to breast cancer as 1) alcohol intake, 2) breastfeeding, 3) diet/nutrition, 4) healthy weight, and 5) physical activity. We defined measures of breast cancer burden as 1) mortality, 2) incidence, and 3) prevalence of a timely mammography.

**Table 2:** Summary of Breast Cancer–Related Recommendations and Guidelines Published by the US Preventive Services Task Force (USPSTF) and the Centers for Disease Control and Prevention (CDC)^a^

Topic	Population	Recommendation
**USPSTF**		
Alcohol intake (11)	Adults and adolescents	Screening for unhealthy use of alcohol.
Breastfeeding (12)	Pregnant women, new mothers, and their children	Provide interventions during pregnancy and after birth to support breastfeeding. Grade: B
Chemoprevention (7)	Women aged ≥35 y at increased risk for breast cancer	Offer to prescribe risk-reducing medications, such as tamoxifen, raloxifene, or aromatase inhibitors. Grade: B
	Women aged ≥35 y not at increased risk for breast cancer	Do not routinely use risk-reducing medications, such as tamoxifen, raloxifene, or aromatase inhibitors. Grade: D
Genetic risk assessment, testing, screening (8)	Women with a personal or family history of breast, ovarian, tubal, or peritoneal cancer or who have an ancestry associated with *BRCA 1/2* gene mutations	Assess with an appropriate brief familial risk assessment tool. Grade: B
	Women whose personal or family history or ancestry is not associated with potentially harmful *BRCA 1/2* gene mutations	Do not perform routine risk assessment, genetic counseling, or genetic testing. Grade: D
Healthy weight (13)	Adults with a body mass index ≥30	Offer or refer to intensive, multicomponent behavioral interventions. Grade: B
Mammogram (14)	Women aged 40–49 y	The decision to start screening should be an individual one. Grade: C
	Women aged 50–74 y	Screen every 2 years. Grade: B
	Women aged ≥75 y	No recommendation. Grade: I (Insufficient evidence)
**CDC**		
Alcohol intake (15)	—	CDC recommends drinking alcohol in moderation and refers to the Dietary Guidelines for Americans for recommendations.
Breastfeeding (16)	Pregnant women	Recommend exclusive breastfeeding for 6 months, and then continuing breastfeeding while introducing complementary foods until the baby is aged 12 months or older
Chemoprevention (17)	Women at high risk of breast cancer	Prescribe aromatase inhibitors, tamoxifen or raloxifene.
Diet/nutrition (18)	—	CDC extensively discusses the topic and refers to Dietary Guidelines for American.
Genetic risk assessment, testing, screening (19)	Women at high risk of breast cancer	CDC extensively discusses the topic but does not provide any guidelines.
Healthy weight (20)	—	CDC extensively discusses the topic and refers to Dietary and Physical Activity Guidelines for Americans for recommendations but does not provide any guidelines.
Mammogram (21)	—	CDC extensively discusses the topic but does not provide any guidelines.
Physical activity (22)	Preschool-aged children (aged 3–5 y)	Every day throughout the day.
	Children and adolescents (aged 6–17 y)	1 hour or more of moderate-to-vigorous intensity physical activity daily.
	Adults (aged 18–64 y)	At least 150 minutes per week of moderate-intensity activity. At least 2 days per week of activities that strengthen muscles.
	Older adults (≥65 y)	At least 150 minutes per week of moderate-intensity activity. At least 2 days per week of activities that strengthen muscles. Activities to improve balance.

a This table was designed by the authors to enable a study of breast cancer–related content in the Comprehensive Cancer Control plans in 50 states and the District of Columbia, January–March 2019.

We then summarized the breast cancer prevention recommendations and guidelines issued by the USPSTF, including only recommendations with an A or B rating (7,8,11–14), and CDC (15–22) (Table 2). Both CDC and USPSTF addressed mammograms; breastfeeding; genetic risk assessment, testing and screening; healthy weight; chemoprevention; and alcohol use. CDC addressed all general cancer prevention topics that apply to breast cancer and referred to the dietary guidelines from the US Department of Agriculture and the US Department of Health and Human Services (3). The USPSTF also has recommendations for weight loss to avoid obesity (13).

**SMART objectives**. To evaluate written objectives (10) we used CDC guidelines for objectives that are SMART: specific, measurable (baseline and target), attainable/achievable (target setting method), relevant (with data source), and time-bound (dated). Specific objectives refer to a particular topic, for example, in our study, mortality caused by breast cancer. Measurable objectives are made concrete by quantification: How will the objective be measured? Achievable targets are often based on history (eg, performance in the previous 5 years) or outcomes achieved by others (eg, median of all states) or are aspirational (eg, “best”). Objectives are most useful when oriented to attainable targets. We did not address “attainable/achievable” unless it met a USPSTF recommendation or CDC guideline. Relevant objectives have an existing, accessible, and specific source of data. As an example, all states have data collection systems for collecting and reporting cancer mortality, incidence, and screening prevalence. We considered both collectors of data (primary sources such as an incidence registry) and users who publish reports (secondary sources) to be data sources. We defined “time-bound” as the baseline date and end date of the CCC plan and refer to it as “time period specified” in this study.

**Abstraction of content from CCC plans**. We used the following search terms to locate content relevant to breast cancer in each CCC plan: alcohol, BRCA, breast, breast cancer, breastfeeding, chemotherapy, chemoprevention, diet/nutrition, drink, family history, food, fruit, gene, genetic, hereditary, obese, obesity, physical activity, mammogram, mammography, nutrition, screening, vegetable, and weight.

To assess the extent of coverage of measures, we created a standardized form to abstract plan content by dichotomous (yes/no) assignment in Excel (Microsoft Corporation) based on the criteria listed in the CDC and NCI cancer control plan development and assessment tool; the form also included a notation on topic or data with source and date and relevant SMART objective, where applicable (10). In the Excel spreadsheet, we assigned breast cancer–related topics and objectives extracted from CDC and USPSTF recommendations and guidelines to columns, and US states and the District of Columbia to rows. We revised the abstraction strategy several times based on the content obtained from CCC plans we considered a priori of high quality. By using the key term search feature of Microsoft Edge, we located plan content in any part of the CCC plan (eg, background information, information related to objectives), classified content into dichotomous variables, evaluated the key term, and then evaluated the surrounding text of the key terms for relevance to breast cancer in all plans. We quantified a dichotomous (yes/no) assignment by using the “count if” feature of Excel, and we spot checked assignments manually to ensure quality. We ascertained date and source of baseline measures and categorized them as present or not present.

We assessed topics in 2 ways: 1) whether the topic (yes/no) was discussed in the CCC plan and 2) whether a topic-specific objective was stated (yes/no). We quantified the findings in Excel as percentage of state plans covering each topic.

## Results

Of the 51 CCC plans, 71% (n = 36) presented data on breast cancer incidence and 76% (n = 39) presented data on mortality (Table 3). Most plans complied with the CDC data quality requirement by including information on the date and source of data. The placement of this information varied among plans: next to these data, in the text, in figures or tables, or at the end of the plan in the plan’s list of references. A few plans referenced other reports rather than citing the primary data source and date. Although data on breast cancer incidence and mortality were commonly included in plans, 12 plans did not include these data. Two states and the District of Columbia presented data on the geographic distribution of breast cancer incidence and mortality across wards (in the District of Columbia) or counties. About half (n = 27) of the plans presented data on the prevalence of a timely mammography.

**Table 3:** Comprehensive Cancer Control Plans That Included Data on Breast Cancer Incidence and Mortality and the Prevalence of a Timely Mammogram and Specified SMART Objective Components^a^, 50 States and the District of Columbia^b^

Topic	Measure			SMART Component^b^				
	Discussed in Plan	Dates Are Specified for Data	Sourced	Specific Objective	Measured Baseline	Measured Target	Relevant Data Source	Time Period Specified
Incidence	36 (71)	34 (67)	25 (49)	1 (2)	1 (2)	1 (2)	1 (2)	1 (2)
Mortality	39 (76)	37 (73)	27 (54)	12 (23)	11 (21)	11 (21)	10 (19)	12 (23)
Prevalence of a timely mammogram^c^	25 (49)	29 (57)	34 (67)	43 (85)	41 (81)	40 (79)	33 (65)	34 (67)

a SMART objectives are specific, measured, attainable/achievable (not assessed in this study), relevant data, with the time specified (10).

b Study was conducted January-March 2019. All values presented are number (percentage).

c Per US Preventive Services Task Force recommendations (14).

Components of SMART objectives were included infrequently for breast cancer incidence (2%; n = 1) and mortality (19%–23%; n = 10–12). When mammography objectives were presented, they usually referred to each SMART component; components least often mentioned were relevant data source (65%; n = 33) and time period specified (67%; n = 34) (Table 3).

Nineteen plans discussed hereditary breast cancer; 14 discussed genetic screening for *BRCA 1/2* mutations. Three plans discussed chemoprevention for women at high risk of breast cancer, and 1 of these CCC plans specified high-risk breast cancer target populations. Uniformly, when chemoprevention was included, the CCC plans did not indicate breast cancer–specific SMART objectives.

Other breast cancer–specific prevention topics were covered to a varying extent in background information. Many plans provided data on these topics in their discussion of baseline rates, prevalence, or whether an objective was met or not in the background section. Five plans discussed breastfeeding as a primary prevention strategy, without any state-specific data (Table 4).

**Table 4:** General Cancer Prevention Topics That Apply to Breast Cancer in Comprehensive Cancer Control Plans, 50 States and the District of Columbia^a^

General Prevention Topics	Topic Included	Stated Link to Breast Cancer	Component of SMART Objective^b^				
			Specific Objective	Measured Baseline	Measured Target	Relevant Data Source	Time Period Specified
Alcohol intake	11 (21)	10 (20)	10 (20)	10 (20)	10 (20)	8 (15)	7 (14)
Breastfeeding	5 (10)	5 (10)	5 (10)	5 (10)	5 (10)	4 (8)	5 (10)
Diet/nutrition	20 (39)	6 (12)	29 (57)	24 (47)	23 (45)	20 (39)	24 (47)
Healthy weight	31 (60)	22 (43)	36 (71)	35 (69)	32 (63)	31 (61)	27 (53)
Physical activity	22 (43)	10 (20)	31 (60)	27 (53)	26 (52)	22 (43)	26 (51)

a Study was conducted January through March 2019. All values presented are number (percentage).

b SMART objectives are specific, measured, attainable/achievable (not assessed in this study), relevant data, with the time specified (10).

Approximately 39% to 60% (n = 20–31) of plans covered 5 general cancer prevention topics that apply to breast cancer (alcohol intake, breastfeeding, diet/nutrition, healthy weight, and physical activity) (Table 4); fewer plans (n = 6–22) discussed the link between these factors and breast cancer. Alcohol intake was addressed in 11 plans; 10 states stated an alcohol-related objective. Specific SMART objectives on healthy weight, physical activity, and nutrition were included in 29 to 36 plans, most often presented with SMART objectives. In addition, a state’s objective targets were presented in most plans, but only 3 plans described the methods for setting goal (target) amounts for their objectives, and of these 3 only 1 plan described methods for every general cancer prevention objective. Some plans used Healthy People 2020 targets or specified a percentage improvement.

## Discussion

Not all 51 CCC plans discussed CDC and USPSTF guidelines and recommendations, and at least half of the plans covered only 4 of the 8. Not all plans addressed SMART objectives, despite CDC’s recommendation to include SMART objectives. Our findings on breast cancer from CCC plans may be transferable and beneficial to planning for other types of cancer to reduce cancer burden state by state and ultimately provide a state planning example to other spheres of public health.

Omission of data on breast cancer mortality in CCC plans was unexpected, because every state and the District of Columbia has agency over their death data. Central cancer registries are a more recent source of data, and inclusion of incidence data was almost as common as inclusion of mortality data, which suggests that data on mortality and incidence may be coming from the same agency source or that states’ understanding of cancer burden encompasses both incidence and mortality data. Mammography data, however, which can be obtained from CDC’s Behavioral Risk Factor Surveillance System surveys (“Women aged 50–74 who have had a mammogram within the past two years”) that are conducted at the state level and are publicly available, were included approximately half the time in the CCC plan background sections and were just as often included as an objective baseline elsewhere. Neglecting to highlight mammography in background information is potentially detrimental to states’ efforts to decrease breast cancer mortality, because screening accounted for more than one-quarter of the decline in breast cancer mortality (5,6) in the past 10 to 25 years. Presenting mammography rate as background information highlights its importance as a public health intervention and can increase survival rates through early detection.

Other topics were discussed in plans to a modest extent: healthy weight (60%), physical activity (43%), nutrition (39%), hereditary breast cancer (37%), testing for *BRCA 1/2* gene mutations (27%), alcohol (21%), breastfeeding (10%), and chemoprevention (6%). Specifying the role of *BRCA 1/2* was less frequent than including the more general topic of hereditary breast cancer. We might expect that these 2 topics would be covered similarly because, as targets for breast cancer prevention, *BRCA 1/2* gene mutations are a subset of heredity breast cancer, although the role of additional genes is becoming more evident over time. We found no SMART objectives for these recommendations. Only 1 in 5 plans specified alcohol use as a risk factor and only 1 in 10 plans specified breastfeeding as a preventive factor. These omissions are surprising, given the evidence base for each. Alcohol use is a modifiable breast cancer risk factor, even at 1 drink per day, and thus even moderate risk projections of breast cancer occurrence can be lowered with abstinence (23). Breastfeeding is also a modifiable factor; the risk of developing breast cancer decreases 4.3% for each year of breastfeeding (24). These preventive factors have not been customary targets of cancer prevention and control programs and will demand work with public health partners across domains and less single-focus thinking about what can be done to enhance cancer prevention.

In general, the topics of primary and secondary prevention of breast cancer and conformance to SMART objectives were mentioned in many, but not all, state CCC plans. For states without such content, a review of the epidemiology literature or a compendium of authoritative recommendations and guidelines for breast cancer prevention would inform and perhaps encourage including them in CCC plans. Surveilling changes in guidelines and recommendations and the use of SMART objectives are additional and valuable feedback to states’ cancer prevention and control efforts as their CCC plans are updated. The inconsistent inclusion of evidence-based primary and secondary prevention recommendations and guidelines as SMART objectives in state CCC plans suggests that CDC, as the funder of state CCC plan development, may need to provide more guidance and technical support on these topics, including information on best practices that illustrate the practical benefit of inclusion. Some resources currently provided at the CDC website are the Cancer Plan Self-Assessment Tool (10), Nutrition and Physical Activity Strategies for Cancer Prevention (25), and Principles for Community Engagement (26). For transparency and validity reasons, developers of state plans need to improve the identification, use, and citation of authoritative sources of data on breast cancer incidence, breast cancer mortality, and mammography prevalence. CDC may need to provide additional guidance and technical support to encourage state plan developers to engage with public health professionals who are familiar with these data sources in their own states or with databases available through resources such as the National Cancer Institute’s Cancer Control Planet State Profiles (26).

In reviewing the 51 CCC plans, we noted features in some that may be useful for implementation of plan objectives. For example, 3 states presented maps of breast cancer incidence and mortality. In studies of US county data, outcomes differ among counties and are influenced by characteristics such as urbanization or population demographics (26,27).

Other sources of variation in breast cancer data are age at diagnosis, race, and ethnicity. Breast cancer diagnosis peaks in the 60s and 70s (26,27). Age at diagnosis is critically informative in planning for breast cancer survivorship among the more than 250,000 women in the United States annually who survive breast cancer (1). Moreover, race/ethnicity and age have traditionally segmented risk status for breast cancer incidence and survival. Median age at diagnosis is a few years younger for non-Hispanic Black women, partly because their rate of triple negative breast cancer, which occurs at younger ages, is twice that of other racial/ethnic subgroups (26,27). Information on age at diagnosis among diverse populations is helpful for setting state-specific subgroup screening guidelines. More importantly, subgroup identification is necessary to address and ultimately achieve equity in outcomes.

Among topics specific to breast cancer, screening by mammography is well covered in CCC plans, especially in the section on objectives. Effectiveness of breast cancer screening in decreasing breast cancer mortality is supported by scientific literature (5,6), and new evidence, such as the evidence provided in our study, contributes to new or amended recommendation statements. Differences between guidance and state objectives may be due to the publication date of the plan predating the latest recommendation or guideline (28). One solution is to revise state CCC plans on a periodic schedule short enough to ensure that new information is incorporated in a timely way.

Breastfeeding, chemoprevention, and hereditary breast cancer barely covered SMART objectives in CCC plans. However, they are included in recommendations and guidelines. Absence in the plans may be due to factors such as lack of awareness among program staff, their smaller effect on risk of developing breast cancer, or a relatively small target population. Their absence further emphasizes the importance of regular staff training, academic and clinical partnerships, and formal specification of recommendation statements in state intervention programs. Anecdotally, state funding may be a single-focus issue in that state programs do not interact with other key programs even when common issues exist (eg, maternal and child health, breastfeeding).

Although the topics of physical activity (22), healthy weight (20), and diet/nutrition (18) are covered extensively on CDC websites and discussed in the guidelines of the US Department of Agriculture and the US Department of Health and Human Services (3), alcohol intake guidelines (15,23) are not frequently covered in CCC plans. CDC may need to conduct educational programs about this lifestyle behavior risk.

State plans vary in their length and style, which may indicate that the plan writers are trying to reach various audiences. CDC may be able to assist with assembling data and plans for these audiences, as the agency is already doing with data visualizations (1).

Our study has several limitations. First, our assessment did not cover all topics recommended by CDC in state CCC plans. For example, CDC recommends discussing information on state demographic factors (10), but it does not specify discussion of these factors in relation to breast cancer recommendations, even though breast cancer has been linked epidemiologically to age, race, ethnicity, income, education, and other factors. Knowledge of these demographic factors is important in designing and carrying out a plan that addresses diversity and ensures equity (29,30). Second, we reviewed the CCC plans available at a single point in time. The periods during which the plans were assembled and intended to serve varied; states having the most plan editions will most likely also be the most complete in the inclusion of topics and use of SMART objectives. In a study of the guidelines and recommendations in the Maryland CCC plan, Fowler and colleagues found that 9 of 19 cancer-related CDC guidelines or USPSTF recommendations had not been issued at the time of 2010–2015 plan publication (28). An ongoing process of reviewing national guidelines and recommendations and updating state CCC plans is needed, especially when the time span of the state’s cancer plan is lengthy (>5 y) or occurs when the next plan is revised.

The evaluation of SMART objectives in our study may have implications for other health planning in the nation, states, or localities (31,32). The highest priorities of the Association of State and Territorial Health Officials for State Health Improvement Plans include assembling data and writing objectives (31). Use of SMART objectives gives all partners a common lexicon, an expectation of achievement over time, and clarity on the path to success. SMART objectives, when complete, are associated with improved outcomes (30).

Our examination of breast cancer–related evidence-based coverage and completeness of plan objectives in state CCC plans shows there is room for improvement. Our findings can guide efforts to improve the quality of all CCC plan topics. However, CDC guidance alone may not be enough to ensure a high-quality plan in every state. In addition to raising awareness of evidence-based planning, other measures may be needed to incentivize best practices in cancer prevention and other areas of public health planning.
